# Challenges and solutions for the analysis of *in situ*, *in crystallo* micro-spectrophotometric data

**DOI:** 10.1107/S1399004714015107

**Published:** 2015-01-01

**Authors:** Florian S. N. Dworkowski, Michael A. Hough, Guillaume Pompidor, Martin R. Fuchs

**Affiliations:** a Swiss Light Source, Paul Scherrer Institute, CH-5232 Villigen PSI, Switzerland; b School of Biological Sciences, University of Essex, Wivenhoe Park, Colchester CO4 3SQ, England; c European Molecular Biology Laboratory Hamburg, c/o DESY, Notkestrasse 85, D-22603 Hamburg, Germany; d Photon Sciences, Brookhaven National Laboratory, Mail Stop 745, Upton, NY 11973, USA

**Keywords:** micro-spectrophotometer, UV–visible absorption, Raman spectroscopy, single-crystal spectroscopy, software toolbox, data analysis, background correction

## Abstract

Combining macromolecular crystallography with *in crystallo* micro-spectrophotometry yields valuable complementary information on the sample, including the redox states of metal cofactors, the identification of bound ligands and the onset and strength of undesired photochemistry, also known as radiation damage. However, the analysis and processing of the resulting data differs significantly from the approaches used for solution spectrophotometric data. The varying size and shape of the sample, together with the suboptimal sample environment, the lack of proper reference signals and the general influence of the X-ray beam on the sample have to be considered and carefully corrected for. In the present article, how to characterize and treat these sample-dependent artefacts in a reproducible manner is discussed and the *SLS-APE* *in situ*, *in crystallo* optical spectroscopy data-analysis toolbox is demonstrated.

## Abstract 2

The particular challenge of the analysis of optical absorption and Raman spectroscopic data measured from protein crystals and how the *SLS-APE* software toolbox supports scientists in dealing with such data is described.

## 1. Introduction   

Complementary biophysical techniques are a powerful tool to complete and enhance structural information obtained by macromolecular crystallography (MX). The structural information provided by electron-density maps obtained from diffraction experiments often does not provide sufficient insight to answer the point of interest conclusively. This is especially true in the fields of enzyme kinetics and protein function, ligand-binding modes or redox states of metal centres. *In situ* micro-spectrophotometry is a highly effective way to obtain such complementary information about the chemical identity of cofactors, the electronic state of metal centers or about specific radiation-induced chemistry. Since its first introduction in the 1970s (Rossi & Bernhard, 1970 ▶) it has come a long way, and today is implemented in several experimental endstations of macromolecular crystallography beamlines at storage rings around the globe, allowing a larger community of structural biologists to benefit from the opportunities provided by this complementary method. Examples include instruments at the ESRF (Carpentier *et al.*, 2007 ▶; Royant *et al.*, 2007 ▶; Davies *et al.*, 2009 ▶), APS (Pearson *et al.*, 2007 ▶), NSLS (Stoner-Ma *et al.*, 2011 ▶; Orville *et al.*, 2011 ▶), DLS (Allan *et al.*, 2013 ▶), SPring-8 (Sakai *et al.*, 2002 ▶; Shimizu *et al.*, 2013 ▶) and SLS (Owen *et al.*, 2009 ▶; Pompidor *et al.*, 2013 ▶). Spectroscopies successfully applied to protein crystals *in situ* include UV–visible absorption, fluorescence, resonance Raman and non­resonance Raman as well as X-ray absorption spectroscopy. A particularly useful application of complementary techniques is the assessment of X-ray-induced photophysics in the sample, also known as radiation damage. The combination of *in situ* spectroscopies with X-ray-induced photophysics allows the characterization of transient states accessible by electron transfer (Schlichting *et al.*, 2000 ▶; Hough *et al.*, 2008 ▶; Adam *et al.*, 2009 ▶; Hersleth & Andersson, 2011 ▶), monitoring of the oxidation states of metal centres (Beitlich *et al.*, 2007 ▶; Ellis *et al.*, 2008 ▶; He *et al.*, 2012 ▶; Merlino *et al.*, 2013 ▶), quantification of radiation damage (Meents *et al.*, 2007 ▶; Rajendran *et al.*, 2011 ▶; McGeehan *et al.*, 2007 ▶) and the general understanding of its mechanisms (Carpentier *et al.*, 2010 ▶; Sutton *et al.*, 2013 ▶).

While synchrotron users are often proficient in interpreting solution spectroscopic data, the particular artefacts observed in the spectra of samples in crystalline form can be easily misinterpreted. Spectroscopic measurement series typically have to cover multidimensional parameter spaces, and a rapid overview of the collected data is paramount in making maximum use of precious beamtime. The provision of a freely available spectroscopic data-analysis toolbox will therefore be a useful aid for users to properly process and gain the maximum biochemical information from their *in crystallo* spectroscopic data and overall crystallographic experiment.

### 1.1. UV–visible absorption spectroscopy   

**Figure 1:**
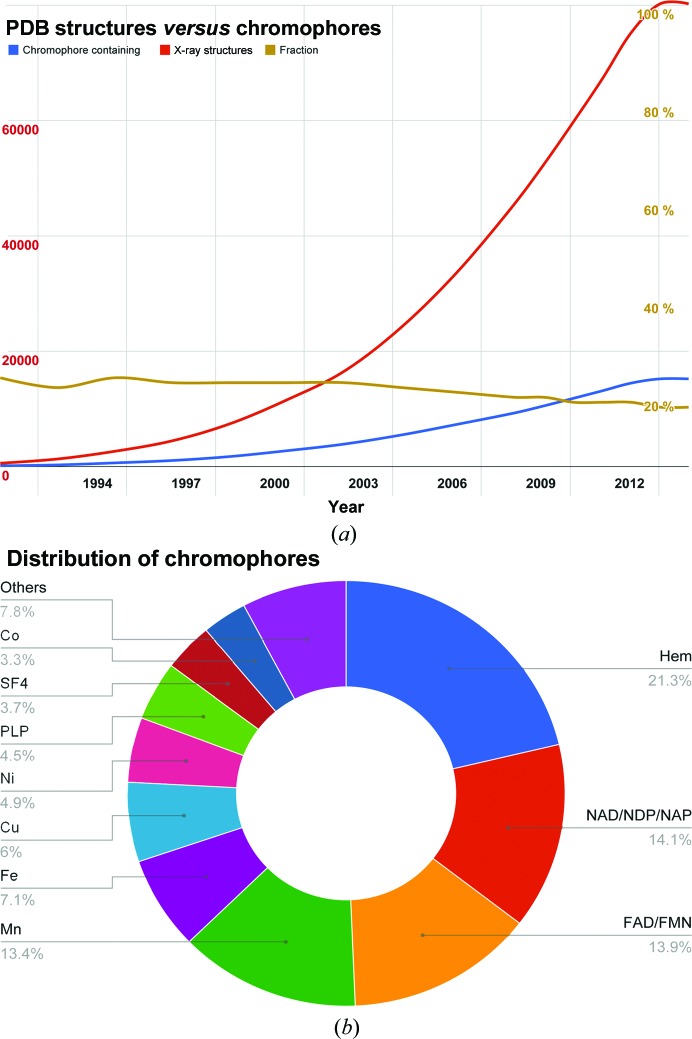
(*a*) Relationship between the total number of X-ray structures deposited in the PDB (red) and structures containing chromophores (blue). The fraction of chromophore-containing structures (yellow) shows only a minimal decrease over this period. (*b*) Distribution of chromophores in X-ray structures deposited in the PDB containing common chromophores as of January 2014.

Absorption spectroscopy has been widely applied to fingerprint the states of chromophores in protein crystals and to monitor changes in these during diffraction experiments. This method is particularly suitable where a relatively low spectroscopic sensitivity but a high time resolution is required. The main disadvantage of the method is the fact that it not only requires the presence of a chromophore in the sample but also that this chromophore is affected by the reaction under observation. A large fraction (roughly 20%) of all of protein structures deposited in the Protein Data Bank (PDB) contain a chromophore (Orville *et al.*, 2011 ▶). Based on the set of most common chromophores defined by Orville and coworkers, one can estimate this fraction to have remained more or less constant over the exponential growth of the PDB in recent years (Fig. 1 ▶ and Supporting Information). In summary, approximately one fifth of all PDB structures are suitable in principle for absorption spectroscopy. However, only a very small proportion of those samples have been investigated spectroscopically. The potential of the additional information to be gained here is the main motivation behind the efforts of researchers and the PDB to include the possibility of submitting spectroscopic data to the PDB alongside the structural model (Garman & Weik, 2011 ▶; Orville *et al.*, 2011 ▶).

### 1.2. Raman and resonance Raman spectroscopy   

In contrast to absorption spectroscopy, Raman spectroscopy (RS) does not require the presence of a chromophore. Instead, any bond vibration of proper symmetry can transfer or receive energy to or from a scattered photon, theoretically allowing most bonds in the molecule to be probed. While this is in general an advantage, it can become a problem in the case of complex molecules such as proteins, since the wealth of information makes the interpretation of the resulting spectra a challenging task. The maximal theoretical number of vibrational modes for a nonlinear molecule can be computed by the formula 3*n* − 6, where *n* denotes the number of atoms present. In a crystal one has to consider not only the single molecule but the number of molecules in the unit cell owing to the non-isotropic orientation. In the case of the tetragonal trapezoidal hen egg-white lysozyme (HEWL; PDB entry 1lyz; Diamond, 1974 ▶), which contains eight molecules in the unit cell, this results in 46 992 possible modes. Even though not all possible vibrational modes are Raman active, it is near-impossible to perform *de novo* peak assignment without additional information, for example that gained from isotope-labelling experiments.

However, it is possible to selectively excite bond vibrations related to a chromophore by utilizing light of a wavelength corresponding to the absorption band of that chromophore (Harrand & Lennuier, 1946 ▶). This so-called resonance Raman (RR) effect makes the measurement and interpretation of protein spectra significantly easier, as bands arising from the chromophore will be of greatly increased intensity (by several orders of magnitude) and far fewer in number than bands measured in nonresonance experiments. However, owing to the higher energy absorption upon photo-irradiation this technique may also have destructive effects on the sample. Thus, great care has to be taken in the balance between photo-excitation and photo-emission to avoid sample alteration or bleaching of the sample. The use of both methodologies for protein analysis has been well described (Rippon *et al.*, 1971 ▶; Spiro & Strekas, 1974 ▶; Carey, 1978 ▶, 1999 ▶; Palings *et al.*, 1987 ▶; Thomas, 1999 ▶). The clear disadvantage of the RR technique compared with RS is the requirement for a chromophore within the protein crystal.

## 2. Limitations of *in crystallo* spectroscopy   

### 2.1. Protein crystals as samples and the nature of *in crystallo* spectra   

In addition to the limitations of the techniques described above, optical spectroscopy on solids is in general no easy task. For absorption techniques the samples must be thin and transparent enough to pass light. Depending on the absorption coefficient of the chromophore in question, even a 10 µm thick plate-like crystal might be too thick. Often the crystal is surrounded by a liquid or amorphous solid phase consisting of the crystallization buffer and cryoprotectant. The chemical components of these can contribute to the spectra and have to be carefully subtracted. Owing to the different diffractive indices of the crystal and the liquid, beam displacement might occur, making it difficult to correctly align the sample and amplifying stray light artifacts. Samples should ideally be prepared with as little buffer as possible on a support (mesh or loop) allowing unhindered illumination. Newly available UV-transparent mounting loops (*e.g.* Mitegen UV-Vis Mounts) can further improve the spectral quality. A good way to mount a crystal, in particular for Raman measurements, is to use a loop significantly smaller than the sample so that the crystal sticks out enough to allow unhindered measurements. However, this may not be appropriate for very thin plate-like crystals, which are optimal for absorption spectroscopy, since the surface strain might cause bending.

**Figure 2:**
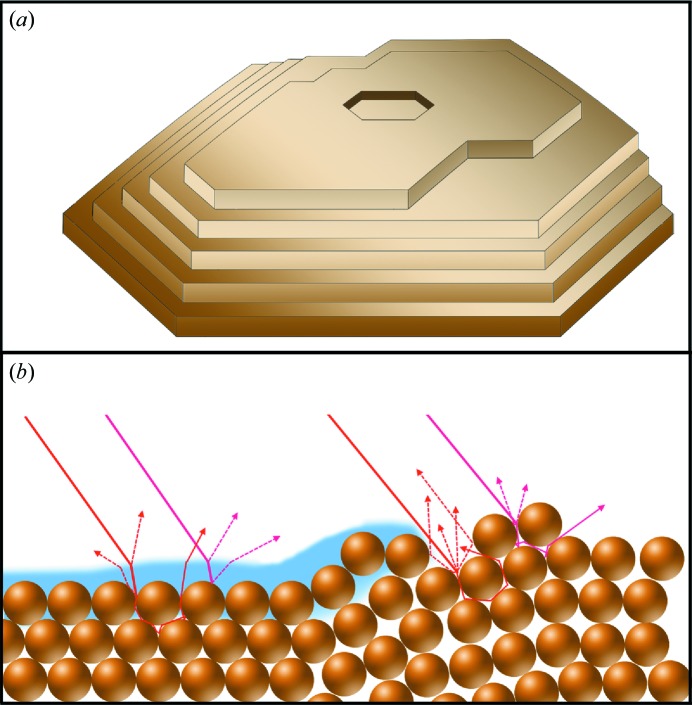
Stray light generation by crystalline samples (see text). (*a*) Grating effect of crystal layers. (*b*) Refraction owing to change in refractive indices and Rayleigh scattering owing to imperfect crystal surface.

Owing to the high scatter density and the imperfect surface structure (*e.g.* crystal layer grating; see Fig. 2 ▶), Rayleigh scattering in solids can be up to 10^6^ times stronger than in solution samples (Julien, 1980 ▶; Ohana *et al.*, 1986 ▶), thus significantly decreasing the signal-to-noise ratio (SNR). Since conventional single-monochromator spectrographs are usually able to attenuate stray light with an efficiency of only about 10^−5^ (Ohana *et al.*, 1986 ▶; Kim *et al.*, 2010 ▶), the use of an additional dichroic filter or a double monochromator is mandatory to achieve a sufficient SNR. It is also critical to find a good alignment of spectroscopic axes towards the sample. This is important to minimize beam displacements on the surfaces in absorption measurements and scattering away from the collection objective in scattering techniques. Usually this has to be achieved by rotating the sample in the instrument until good spectra can be acquired.

Since not only the protein in the crystal but essentially all substances irradiated by the excitation laser can emit Raman photons, contaminants, buffers or ligands can all contribute to the measured RS or RR spectrum. In fact, the resulting background signal can be some orders of magnitude more intense than the weak Raman signal arising from the protein. It is thus desirable to eliminate as many of these factors as possible from the sample. Since this is hardly possible in *in crystallo* spectroscopy, at least the Raman signatures of the individual components should be known or measured independently and subtracted from the measured spectrum. Fortunately, water is a poor Raman scatterer owing to the lack of polarizable bond vibrations, so that, in contrast to infrared spectroscopy, aqueous solutions are usually a better choice compared with organic solvents. Both RS and RR spectroscopies probe the region of the crystal that is penetrated by the excitation laser beam. Under resonance or near-resonance conditions, this penetration depth is of the order of a few micrometres, meaning that there is a major benefit for an on-axis geometry such that the spectroscopically probed region corresponds to a part of the crystal that has been exposed to the X-ray beam during a crystallographic experiment.

We should note that we explicitly do not discuss the proper treatment of polarized Raman or absorption spectra. As the crystal goniometer of our beamline at the Swiss Light Source (SLS X10SA) at present has a single axis, it is not possible to re-orient a crystal along a specific axis. Our micro-spectrophotometer is therefore at present implemented without polarization analyzer stages. As a residual degree of polarization owing to the fibre optics cannot be ruled out, for Raman spectra relative comparisons of vibration-band amplitudes have to be undertaken with the crystal in a specific orientation only (Carpentier *et al.*, 2007 ▶).

### 2.2. Raman band sharpening and shifting   

Raman spectra of crystals can exhibit several distinct differences compared with the respective solution spectra of the same molecule. In solution, the molecules are subject to constant movement, resulting in frequent intermolecular collisions and leading to significant peak broadening. In contrast, the defined arrangement of molecules in the crystal lattice results in a defined set of intermolecular interactions and thus a narrower Raman bandwidth (Gouadec & Colomban, 2007 ▶). This effect is even more pronounced if the sample is cooled, as is often the case in macromolecular crystallography. On the other hand, the tight arrangement in the crystal causes the intramolecular bond vibrations to be influenced by the neighbouring molecules in the crystal, possibly resulting in peak shifting compared with the equivalent solution spectra. Furthermore, vibrations in adjacent molecules in a crystal can either be in phase or out of phase, possibly resulting in peak splitting or intensity fluctuations owing to harmonic amplification or destruction (Hendra, 2002 ▶). The direct consequence of this is that the *in crystallo* spectra can significantly differ from solution spectra and one has to be aware of this during data interpretation.

### 2.3. Fluorescence background in Raman spectroscopy   

The major practical drawback of Raman spectroscopy is the intrinsic background, usually attributed to fluorescence, which can be up to 10^11^ and 10^3^ times more intense than the actual nonresonance Raman or resonance Raman scattering, respectively (Asher, 2001 ▶). This has long been a hindrance in the application of Raman spectroscopy as an effective analytical tool in biology (Rippon *et al.*, 1971 ▶). In proteins it is particularly easy to excite unwanted fluorescence from, for example, aromatic amino acids, buffers or cryoprotectants, or most commonly impurities and contaminants. To minimize these effects, the protein sample has to be of the highest possible purity before crystallization and, where possible, a careful choice of spectroscopically silent crystallization reagents and cryoprotectants is beneficial. Another approach to minimize the background fluorescence is to change the wavelength of the excitation laser away from the absorption band of the fluorophore. Usually the background is significantly reduced if excitation either in the UV or IR regions is utilized. Owing to the complex and expensive instrumentation required, UV Raman measurements, while extremely useful (Asher, 1988 ▶; Kim *et al.*, 2010 ▶; Oladepo *et al.*, 2012 ▶), are significantly less common than IR Raman measurements. A typical laser wavelength for nonresonance Raman experiments is 785 nm, as affordable high-power diode lasers have become available and the sensitivity of CCD detectors is still high at these wavelengths.

In the case of crystals and other solids it has been suggested that there are also other influences on the measured background; for example, point defects on the surface of crystals have been shown to contribute (Splett *et al.*, 1997 ▶; Kim *et al.*, 2010 ▶). These intrinsic defects are unavoidable for protein crystals, and the resulting background has to be dealt with either experimentally or during post-processing of the spectroscopic data. Several methods have been suggested to reduce the Raman background. The invasive method of photo-bleaching of the fluorophores (Macdonald & Wyeth, 2006 ▶) is neither very effective nor desirable. Other methods include time-gating methods, utilizing the fact that the Raman scattering lifetime is typically 10^−11^–10^−13^ s while the fluorescence lifetime is of the order of 10^−6^–10^−9^ s (Laubereau *et al.*, 1972 ▶; Van Duyne *et al.*, 1974 ▶). This method, however, is not applicable for *in situ*, *in crystallo* Raman measurements, since the required acquisition times are incompatible.

An especially useful technique for experimental background reduction is shifted excitation Raman difference spectroscopy (SERDS; Shreve *et al.*, 1992 ▶; Sowoidnich & Kronfeldt, 2012 ▶). Here, two Raman spectra are recorded under identical conditions but utilizing two different excitation lasers with wavelengths typically ∼0.5 nm apart. While the relatively broad background is not affected by this small shift, the Raman peaks are shifted exactly by the excitation wavelength difference. By differentiating the two spectra, the background can be completely eliminated. The drawback of the method is the additional investment in instrumentation and experimental time, as well as the difficult comparison of the difference spectra with ‘normal’ Raman spectra. On beamline X10SA (SLSpectroLAB) at the Swiss Light Source spectroscopic facility we provide two integrated SERDS laser systems at ∼785 and ∼647 nm and appropriate data-processing algorithms *via* the *SLS-APE* (*SLS Laser Spectroscopy Analysis and Processing Environment*) toolbox (see §[Sec sec4.1]4.1).

## 3. Instrumentation   

Accommodating a micro-spectrophotometer instrument into an existing MX experimental beamline leads to compromising the maximal performance of the instrument. The sample environment is usually crowded and occupied by various other devices such as a liquid-nitrogen cryostat, an X-ray fluorescence detector, a sample microscope, a sample-illumination lamp and a sample changer. To minimize the footprint of the spectrophotometer and to make it available to a broader user base were the main drivers for the integration of the instrument into the new macromolecular crystallography endstation D3 at SLS beamline X10SA (Fuchs *et al.*, 2014 ▶). The resulting micro-spectrophotometer, named MS3 (Dworkowski *et al.*, in preparation) is based on the very successful on-axis MS2 device previously operated in a mount-per-request mode (Pompidor *et al.*, 2013 ▶) and is always in place and available to beamline users. The on-axis geometry helps to ensure good alignment of the X-ray beam and the spectroscopic light path (UV–visible or Raman excitation laser) as well as to ensure that spectroscopic data are measured from the X-ray-exposed volume of the crystal. A detailed description of the on-axis micro-spectrophotometer is available elsewhere (Pompidor *et al.*, 2013 ▶).

## 4. Data analysis   

**Figure 3:**
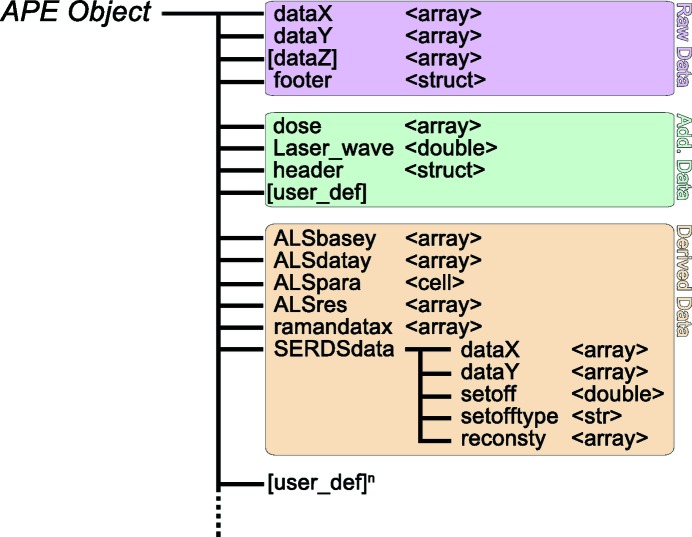
The general structure of an *SLS-APE* data object. The raw data segment is never altered by the toolbox and all derived data can thus be tracked back to the original data set (pink). Additional data can be added depending on the experimental requirements (green). All data derived *via* the toolbox are assigned to dedicated data containers (beige).

As many of the previously mentioned interferences and data artefacts either cannot be avoided or can only be avoided to a limited extent, careful post-processing of the experimental data is imperative. To make this task easier and, more importantly, more reproducible and reliable, we have developed a software toolbox for this kind of data analysis. The *SLS-APE* toolbox is a *MATLAB*-based (The MathWorks Inc., Natick, USA) library of functions and data-handling procedures for automated *in crystallo* data analysis. Raw data are loaded into a defined data object, which is then utilized by all subprocedures. The toolbox is designed in such a way that the original raw data are never unintentionally altered, but instead all derived data are added to the object, allowing a certain level of backtracking. The basic structure of the data object is shown in Fig. 3 ▶. Since the codebase is open source, it is very easy to expand the data object to suit the experiment and analysis at hand. The toolbox is available from the corresponding author upon request.

### 4.1. Capabilities of the toolbox   

The main purpose of the development of the *SLS-APE* toolbox was the ability to analyze *in crystallo* spectroscopic data easily, flexibly and quantitatively. Special emphasis was put on making data comparison between different samples possible. Since most commercial spectrographs utilize a proprietary data format, the universal first step in the workflow is a conversion to the general *SLS-APE* data format, which is also readable by other *MATLAB*-based routines. At the SLSpectroLAB this entails a conversion from the spectrograph’s proprietary software file format (ANDOR Solis, Andor Technology, Belfast, Northern Ireland), but the implementation of other formats is straightforward, in particular if a *MATLAB* module is supplied by the vendor. Depending on the type of spectroscopic data, this step is followed by calibration/conversion of the data. In the case of Raman data this is a calibration to the exact laser excitation wavelength and conversion to Raman shifts. For this purpose a user-friendly GUI is supplied with the toolbox, the *Raman Calibration Tool* (*RaCaTo*). A reference spectrum of acetaminophen, polystyrene, cyclohexane or silicon is used to calculate the exact laser wavelength, which is then in turn used to calibrate all subsequent spectra. This procedure, especially if repeated throughout the experiment, ensures exact spectral calibration even if the laser wavelength varies owing to aging, long runtimes or heating. Interpretation of convoluted or noisy spectra might pose a challenge. To aid the researcher in an initial assessment, the toolbox can perform basic peak-finding procedures as well as approximate band assignment to a standard or user-defined lookup table.

UV–visible absorption or fluorescence kinetic data usually do not require any additional data processing, and the slicing routines of *SLS-APE* can be utilized directly to integrate peak areas and plot changes over time. In the case of Raman spectra, however, a baseline correction is usually necessary. As discussed previously, fluorescence background is a major problem for the weak Raman signal. This background might be further increased if the experiment involves X-ray irradiation, since the fluorescence can be enhanced by the radiation over time (McGeehan *et al.*, 2007 ▶). This also means the background might not be constant and has to be determined and corrected for each single spectrum in a kinetic series. Performing this task manually, as is often performed in Raman experiments, is not only tedious but also extremely error-prone and subject to user bias. Hence, an automated correction is necessary for reliable data interpretation. Many algorithms for Raman background correction have been suggested, including promising ones based on wavelets (Li, 2009 ▶), multiple polynomials (Zhao *et al.*, 2007 ▶), ‘rolling ball’ filtering (Kneen & Annegarn, 1996 ▶), singular value decomposition (SVD; Palacký *et al.*, 2011 ▶) and principal component analysis (PCA; Hasegawa *et al.*, 2000 ▶). However, when evaluating these algorithms we found various problems, such as changing backgrounds owing to an inability to deal with the varying fluorescence owing to X-ray irradiation, lost peaks and negative values. The fastest and most reliable algorithm we tested and therefore implemented in *SLS-APE* is the asymmetric least-squares (ALS) approach (Boelens *et al.*, 2004 ▶; Peng *et al.*, 2010 ▶), a finding supported by other systematic comparisons of correction models (Liland *et al.*, 2010 ▶). Using the ALS algorithm allows fully automatic and reliable correction of very strong and varying backgrounds so that usually no scaling within a kinetic series is necessary. However, it is possible to scale data in *SLS-APE*, for example for the comparison of different samples or positions in a crystal. Further processing depends on the experiment and can include difference spectra calculation, time slices or dose-dependence calculations.

Another method to exclude background fluorescence from Raman spectra is a SERDS measurement. This method does not rely on computational processing of the spectra but determines the background experimentally. The drawbacks here are an effective doubling of the acquisition time, which can be incompatible with kinetic measurements, and the fact that the resulting spectra are difference spectra, making them difficult to compare with non-SERDS reference spectra. To relieve this latter problem, *SLS-APE* offers the capability to convert SERDS spectra to a normal Raman signal using the algorithm suggested by Matousek *et al.* (2005 ▶).

Finally, the toolbox provides mechanisms to extract, combine and export data for subsequent use in other applications.

### 4.2. Automation   

**Figure 4:**
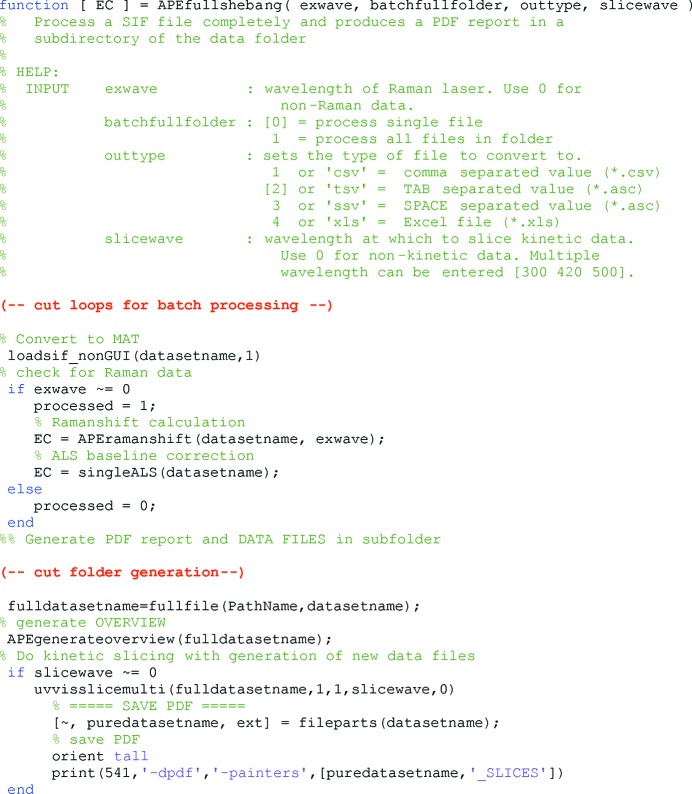
Code example of the *apefullshebang.m* script used at the SLSpectroLAB. This script scans a complete folder of raw spectra and automatically processes them according to the experiment type. Finally, it outputs a PDF overview for the researcher as well as all generated data files in the chosen format.

**Figure 5:**
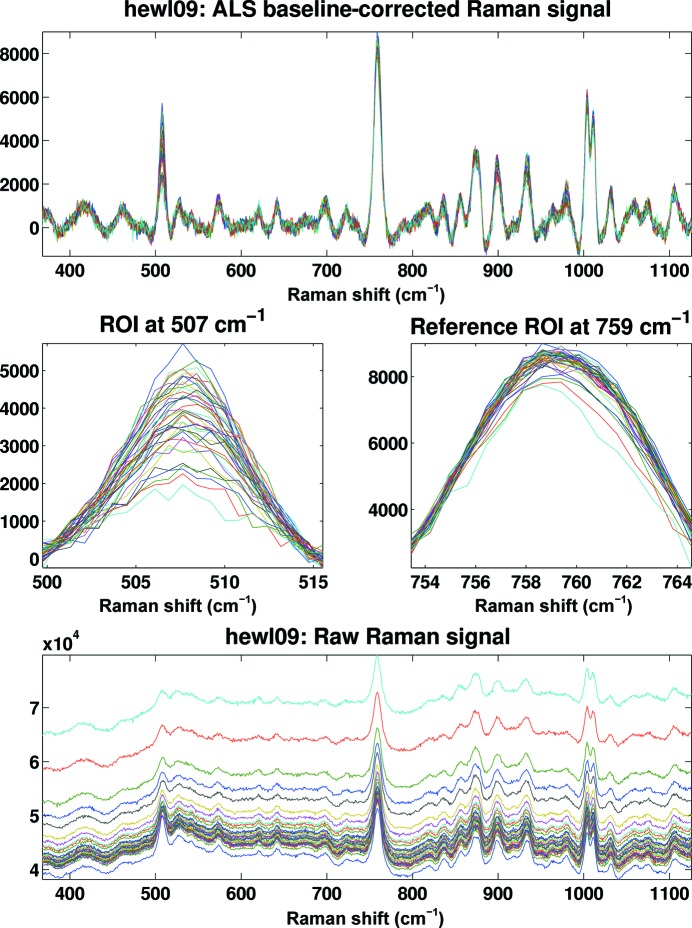
Screenshot of the output of the *SLS-APE* script *ramaninterleave.m*. The raw Raman kinetic data (bottom) are automatically corrected for fluorescence background using the ALS algorithm (top). An invariant peak chosen by the user is then used to normalize the spectra (middle right) and the area under the peak of interest is integrated to plot peak decay against received dose in the subsequent step (middle left). All spectral intensities shown are in counts s^−1^.

An important aspect during the development of the *SLS-APE* toolbox was the possibility of automating the processing of repetitive or complex measurements for convenience, reliability and speed. Hence, all of the tools utilize the same basic data structure and input format. In combination with the powerful plotting capabilities of *MATLAB* it is very easy to design complex analysis scripts. An example is the *apefull­shebang.m* script for Raman spectra processing provided with the toolbox (Fig. 4 ▶). This script is used at the SLSpectroLab after each user shift to quickly convert the data from the proprietary format used by the spectrograph into a universal format (a free choice of comma-separated variable, tab-delimited or Microsoft Excel) to detect and calibrate Raman data and to correct the baseline. In addition, the toolbox can plot an overview sheet in PDF format containing all relevant information and spectra for the user to take home for an initial overview of the acquired data (Fig. 5 ▶). Owing to the quantity of data likely to be collected in a typical 16–24 h shift, this overview can prove crucial in ensuring comprehensive and timely post-processing.

## 5. Case studies   

Two case studies using Raman and absorption data acquired with the micro-spectrophotometer at SLS beamline X10SA (Pompidor *et al.*, 2013 ▶) will be used in the following section to demonstrate typical applications of the *SLS-APE* toolbox.

### 5.1. Case study 1: X-ray energy dependence of S—S bond breakage in lysozyme   

Radiation damage upon X-ray irradiation is a major problem in macromolecular crystallography. Without cryocooling, crystalline samples degrade in the photon beam in a very short time, apparent by loss of high-resolution diffraction spots and overall diffraction power. This time is usually not long enough to measure a complete data set allowing structure solution, although recent developments in high frame-rate detectors hold the promise of a ‘room-temperature renaissance’ (Owen *et al.*, 2012 ▶, 2014 ▶). However, even at cryogenic temperatures of around 100 K the sample will be affected by the radiation, although at a much slower rate (Haas & Rossmann, 1970 ▶; Hope, 1988 ▶; Garman & Schneider, 1997 ▶; Garman & Owen, 2006 ▶). It is thus imperative for the structural biologist to know the extent of damage the sample received during the diffraction experiment to ensure correct interpretation of the structural model.

**Figure 6:**
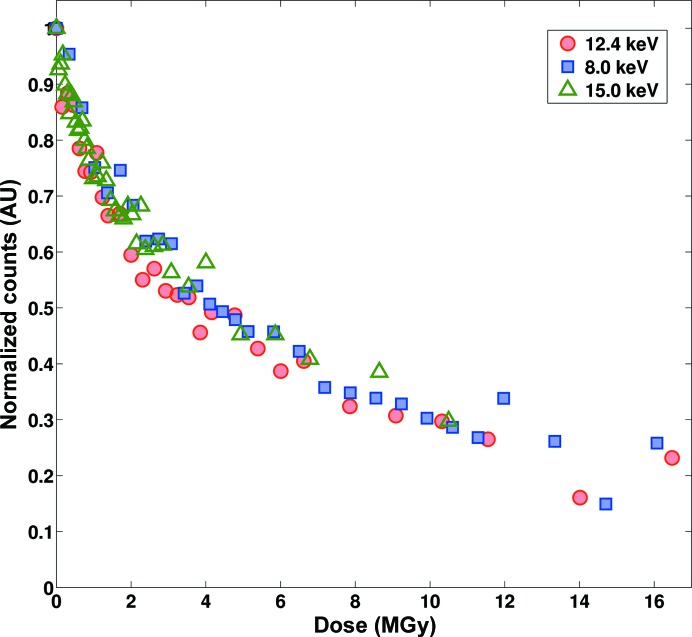
Plot of the decay of the 507 cm^−1^ normalized peak area in a HEWL nonresonance Raman spectrum as a function of received X-ray dose at 8.0 keV (blue), 12.4 keV (red) and 15 keV (green).

Disulfide bridges are commonly found in protein crystals and have been shown to be very sensitive to radiation (Burmeister, 2000 ▶). To demonstrate the usefulness of the *SLS-APE* toolbox in quantifying the dose-dependence of disulfide-bond breakage at different X-ray energies, we performed a stepwise radiation-exposure experiment on hen egg-white lysozyme crystals. The crystals, with average dimensions of approximately 250 × 250 × 250 µm, were irradiated for a defined amount of time at fixed ω with an X-ray beam of 100 × 100 µm at 8.0, 12.4 and 15.0 keV, respectively, followed by the acquisition of a non-resonance Raman spectrum with five accumulations of 20 s acquisition time each. This was repeated with varying X-ray exposure times up to a total dose of >15 MGy for each crystal and X-ray wavelength, allowing a comparison to X-ray doses received by protein crystals during diffraction data collection, which typically lie between 1 and 5 MGy. The resulting spectra were named according to the pattern [spectrum name]_[t###][s###], where [t###] and [s###] denote the beam attenuation and time of exposure, respectively. This information is not contained in the raw spectroscopic file format, but by naming the files in this way the *SLS-APE* toolbox can be used for automated processing of nonstandard experiments. The files are then processed with a simple script in *SLS-APE* consisting of the following steps: (i) conversion of each file to the *SLS-APE* format, (ii) calibration and conversion to Raman shift units, (iii) automatic baseline correction and (iv) calculation and addition of the received dose per exposure by calculation of the equivalent time of unattenuated beam irradiation multiplied by the dose rate calculated using *RADDOSE* (Murray *et al.*, 2004 ▶; Paithankar *et al.*, 2009 ▶), (v) combination of all single data sets of one series into a single array of kinetic data, (vi) integration of the peak area in the region of interest (ROI), (vii) a plot of an overview of the raw and processed data (Fig. 5 ▶) and (viii) a plot of the normalized peak area against the dose received (Fig. 6 ▶).

The resulting overlay of all three measurements shows identical dose dependences for all three X-ray energies within a 95% confidence interval (see Supporting Information), indicating that disulfide-bond breakage is energy-independent, as previously reported (Shimizu *et al.*, 2007 ▶). All three traces fit a previously proposed model in which back-conversion of the anionic radical is significantly accelerated by X-rays, revealing an X-ray-induced ‘repair’ mechanism (Carpentier *et al.*, 2010 ▶).

The data and scripts discussed in this section will be available with the *SLS-APE* toolbox as a tutorial example.

### 5.2. Case study 2: X-ray photoreduction of cytochrome *c*’ from *Shewanella frigidimarina*   

**Figure 7:**
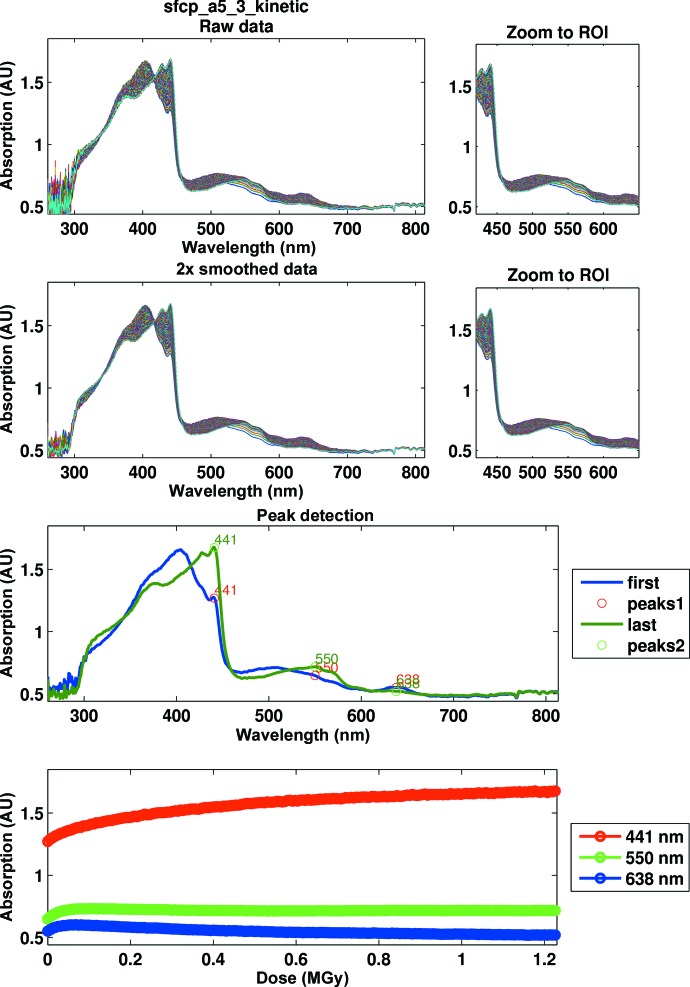
Processing of kinetic data for the X-ray reduction of cytochrome *c*′ from *S. frigidimarina*. The panels in the second row show the smoothed data, those in the third row show the peaks identified in the first and the last spectrum and those in the fourth row show kinetic slices through those peaks. The spectrum of the ferric protein (blue) is rapidly interconverted to that of the ferrous form. Bottom panel: the dose-dependence of absorbance at three spectral peaks. Changes are largely complete after an absorbed dose of 1 MGy.

Cytochrome *c*′ from the marine microorganism *S. frigidimarina* is a haem-containing protein exhibiting pentacoordination of the haem iron with a proximal histidine ligand (Manole *et al.*, unpublished work). Crystals were grown over several days by the hanging-drop vapour-diffusion method. 2 µl 20 mg ml^−1^ protein solution in 20 m*M* Tris–HCl pH 7 was mixed with an equivalent volume of reservoir solution consisting of 0.1 *M* HEPES pH 7, 2.2 *M* ammonium sulfate. A UV–visible absorption spectrum measured on a crystal prior to X-ray irradiation shows a Soret band (406 nm), broad peaks in the α/β region (460–580 nm) and a well defined charge-transfer band (638 nm) consistent with the protein being predominantly in the ferric state (Fig. 7 ▶). The crystal was irradiated with X-rays of 12.4 keV at a dose rate of 15.4 kGy s^−1^ for 80 s, corresponding to a total absorbed dose of 1.23 MGy as calculated in *RADDOSE* (Murray *et al.*, 2004 ▶; Paithankar *et al.*, 2009 ▶), while a kinetic series of UV–visible absorption spectra was recorded. Each spectrum was an accumulation of 20 exposures of 0.002 s, giving a total measurement interval of 0.4 s with a corresponding dose per spectrum of 6.16 kGy. Kinetic data were exported to the *SLS-APE* toolbox and analyzed using a simple script performing the following actions: (i) conversion to the *SLS-APE* data format, (ii) smoothing of the spectra using a Savitzky–Golay algorithm (Fig. 7 ▶, second row), (iii) peak identification in the first and last spectrum of the series (Fig. 7 ▶, third row) and (iv) kinetic time-slices through the changing peaks (Fig. 7 ▶, fourth row). The results are plotted and the derived data as well as the generated figures are automatically saved to the user’s directory.

Upon irradiation, the spectrum corresponding to the ferric form of the protein showed rapid interconversion to a spectrum consistent with the ferrous form of the protein. The Soret band shifted to a split peak (429 and 441 nm) and sharpening of the peaks in the α/β region as well as loss of the charge-transfer peak could be observed. Reduction appeared to be complete after an accumulated dose of 1 MGy.

## 6. Conclusion and future studies   

The *SLS-APE* toolbox has been shown to be effective in aiding researchers in processing, background subtraction and displaying single-crystal spectroscopic data. The toolbox may be applied to UV–visible absorption, fluorescence and both resonance Raman and nonresonance Raman experiments. The application of the best-practice background-subtraction algorithms, together with peak identification and plotting, makes it easier for users to understand the spectroscopic data measured. The high-throughput and automated nature of the toolbox makes it particularly useful in kinetic or otherwise complex experiments where many spectra must be processed in an efficient, reproducible and consistent way. As the current version of the toolbox resulted from a natural evolution of individual analysis tools, it can be optimized for general use.

## Supplementary Material

Supporting Information.. DOI: 10.1107/S1399004714015107/ba5221sup1.pdf
