# Supplementary material for: Challenges and solutions for the analysis of in situ, in crystallo micro-spectrophotometric data
Source: Acta Crystallogr D Biol Crystallogr. 2015 Jan 1;71(Pt 1):27–35. doi: 10.1107/S1399004714015107 (PMC4304683; doi:10.1107/S1399004714015107)
Supplement: Supplementary file 1 [file d-71-00027-sup1.pdf]

BIOLOGICAL
CRYSTALLOGRAPHY

Volume 71 (2015)

Supporting information for article:

Challenges and solutions for the analysis of *in situ, in crystallo* micro-spectrophotometric data

Florian S. N. Dworkowski, Michael A. Hough, Guillaume Pompidor and Martin R. Fuchs

Supporting information

Table S1 Chromophore distribution in X-ray diffraction based PDB structures by year of deposition (as of January 27, 2014).

Chromophore containing	Xray structures	Fraction	Year	PLP	NAD/NDP/NAP	FAD/FMN	Hem	Mn	Fe	FeS	SF4	Co	Ni	Cu	CLA/ CHL	BCA/ BCL	Ret	pterin	Lumi
142	586	24%	1990	11	14	6	75	7	9	1	4	4		10	0	0	1		
292	1319	22%	1992	13	34	28	118	24	17	2	10	11		31	0	2	2		
642	2662	24%	1994	32	61	68	251	69	31	6	34	20	4	58	0	6	2		
1055	4475	23%	1996	48	126	121	354	139	57	12	46	27	12	96	1	9	3	3	1
1739	7416	23%	1998	94	205	181	521	206	123	28	100	42	39	158	2	13	9	17	1
2757	11584	23%	2000	187	321	305	763	318	185	58	135	79	78	204	4	23	25	64	8
3910	16569	23%	2002	260	479	453	1011	471	254	95	180	103	113	278	7	37	48	96	25
5500	24241	22%	2004	340	720	628	1323	699	367	144	245	168	179	357	16	50	74	124	66
7394	34103	21%	2006	431	993	912	1647	966	524	208	300	228	273	459	23	66	91	127	146
9376	45999	20%	2008	543	1289	1224	2035	1231	677	261	345	280	372	566	29	90	102	140	192
10585	52730	20%	2009	576	1473	1428	2245	1374	768	311	384	336	454	632	37	92	115	162	198
11861	59899	19%	2010	599	1643	1635	2553	1531	846	357	440	379	534	715	45	93	123	169	199
13154	67312	19%	2011	634	1859	1800	2795	1718	947	390	486	435	604	814	45	97	140	172	218
14481	75267	19%	2012	668	2052	2022	3041	1917	1043	421	531	478	696	888	52	102	154	181	235
15211	80231	18%	2013	684	2152	2112	3240	2036	1077	436	556	505	748	920	54	108	161	185	237
15213	80250	18%	2014	684	2152	2112	3241	2036	1077	436	556	505	748	921	54	108	161	185	237

IMPORTANT: this document contains embedded data - to preserve data integrity, please ensure where possible that the IUCr Word tools (available from http://journals.iucr.org/services/docxtemplate/) are installed when editing this document.

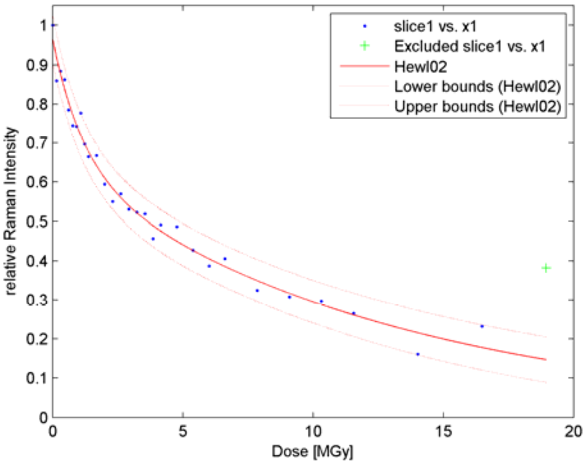


```
General model Exp2:
     f(x) = a*exp(b*x) + c*exp(d*x)
Coefficients (with 95% confidence bounds):
       a =   0.3193   (0.2322, 0.4064)
       b =  -0.8496   (-1.268, -0.431)
       c =   0.645    (0.5543, 0.7357)
       d =  -0.07829  (-0.09676, -0.05982)

Goodness of fit:
    Adjusted R-square: 0.9809
```

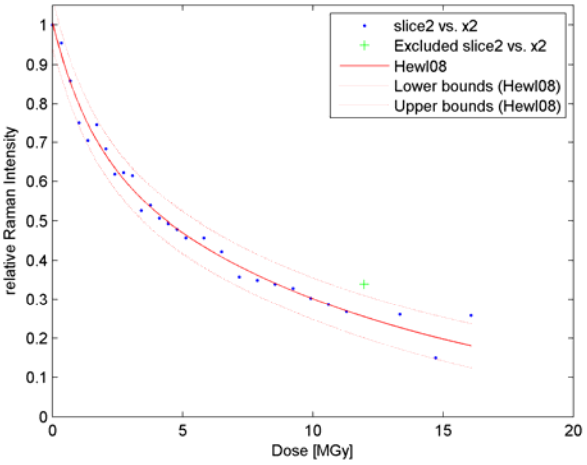


```
General model Exp2:
     f(x) = a*exp(b*x) + c*exp(d*x)
Coefficients (with 95% confidence bounds):
       a =   0.2824  (0.1714, 0.3935)
       b =  -0.7433  (-1.226, -0.2609)
       c =   0.7268  (0.611, 0.8426)
       d =  -0.0893  (-0.1086, -0.06999)

Goodness of fit:
    Adjusted R-square: 0.9839
```

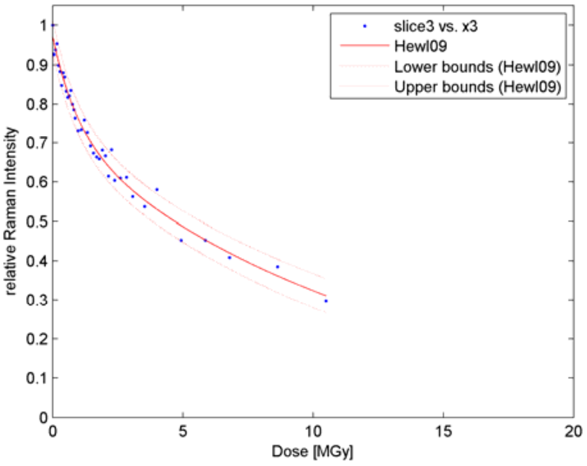


```
General model Exp2:
     f(x) = a*exp(b*x) + c*exp(d*x)
Coefficients (with 95% confidence bounds):
       a =   0.2589  (0.1937, 0.324)
       b =  -0.9611  (-1.349, -0.5736)
       c =   0.7138  (0.6428, 0.7847)
       d =  -0.08017 (-0.09663, -0.06371)

Goodness of fit:
  Adjusted R-square: 0.9871
```

Figure S1 Fit of double exponential to decay data of S-S bonds in HEWL upon X-ray irradiation at 12.4 keV (top), 8.0 keV (middle), and 15.0 keV (bottom). Measured by non-resonance Raman spectroscopy at 785 nm Laser excitation. Decay of peak at 507 cm^{-1} is plotted against received dose. For fitting the Matlab Curve Fitting Toolbox was used.
